# High-Aspect-Ratio Microfluidic Channel with Parallelogram Cross-Section for Monodisperse Droplet Generation

**DOI:** 10.3390/bios12020118

**Published:** 2022-02-14

**Authors:** Hyeonyeong Ji, Jaehun Lee, Jaewon Park, Jungwoo Kim, Hyun Soo Kim, Younghak Cho

**Affiliations:** 1 Department of Mechanical System Design Engineering, Seoul National University of Science & Technology, Seoul 01811, Korea; jhy8718@naver.com (H.J.); kimjw@seoultech.ac.kr (J.K.); 2 Daegu Research Center for Medical Devices and Rehabilitation, Korea Institute of Machinery and Materials, Daegu 42994, Korea; ljh30226@naver.com; 3 School of Microelectronics, Southern University of Science and Technology, Shenzhen 518055, China; jwpark@sustech.edu.cn; 4 Department of Electronic Engineering, Kwangwoon University, Seoul 01897, Korea

**Keywords:** high-aspect-ratio microfluidic channel, parallelogram cross-section, monodisperse droplet, droplet generation

## Abstract

Droplet-based microfluidics has been widely used as a potent high-throughput platform due to various advantages, such as a small volume of reagent consumption, massive production of droplets, fast reaction time, and independent control of each droplet. Therefore, droplet microfluidic systems demand the reliable generation of droplets with precise and effective control over their size and distribution, which is critically important for various applications in the fields of chemical analysis, material synthesis, lab-on-a-chip, cell research, diagnostic test, and so on. In this study, we propose a microfluidic device with a high-aspect-ratio (HAR) channel, which has a parallelogram cross-section, for generating monodisperse droplets. The HAR channel was fabricated using simple and cheap MEMS processes, such as photolithography, anisotropic wet etching, and PDMS molding, without expensive equipment. In addition, the parallelogram cross-section channel structure, regarded as a difficult shape to implement in previous fabrication methods, was easily formed by the self-alignment between the silicon channel and the PDMS mold, both of which were created from a single crystal silicon through an anisotropic etching process. We investigated the effects of the cross-sectional shape (parallelogram vs. rectangle) and height-to-width ratio of microfluidic channels on the size and uniformity of generated droplets. Using the developed HAR channel with the parallelogram cross-section, we successfully obtained smaller monodisperse droplets for a wider range of flow rates, compared with a previously reported HAR channel with a rectangular cross-section.

## 1. Introduction

Droplets have been widely used as biochemical reactors for chemical and biological analysis and templates for polymer microbeads due to their unique features, such as high-throughput, minimal reagent consumption, massive production of droplets, rapid response time, and independent control of each droplet [1,2,3,4,5]. Therefore, droplet microfluidic systems require the reliable generation of droplets with precise and effective control over their size and distribution, which is critically important for various applications in the fields of chemical analysis, material synthesis, lab-on-a-chip, cell research, diagnostic test, and so on.

Compared with conventional droplet production processes, such as mechanical agitation [6] and membrane emulsification [7], methods based on microfluidic channels have several advantages of easy and robust generation of droplets, excellent handling, and monodisperse droplet size ranging from nanometer to micrometer scale [8]. There are various types of microfluidic channel configurations according to the droplet breakup mechanism: T-junction [9,10] and flow-focusing [11] configuration based on shear stress for droplet breakup and step emulsification [12,13], gradient of confinement [14], and HAR (high-aspect-ratio) confinement [15] configuration based on interfacial tension for droplet pinch-off. Using the T-junction structure, fluids with two immiscible phases generate droplets by shear force, whose sizes can be adjusted by changing the relative flow rates of these fluids. In order to generate a large amount of droplets with a uniform size, however, it is necessary to precisely control the flow rate, and thus, microfluidic devices having a precise flow control capability are required. On the other hand, in the step emulsification configuration, the interfacial tension between two immiscible phases by a Laplace pressure difference drives droplet pinch-off when passing through the stepped channel. This droplet breakup mechanism can produce uniform-sized droplets regardless of the interference of flow or pressure fluctuation, enabling massive droplet generation by parallelizing the multiple-step emulsification configurations on a single device. However, one of the drawbacks is that this configuration requires complicated multistep photolithography processes to fabricate stepped channels. Recently, Yao et al. reported a HAR microfluidic channel device that has a similar droplet generation mechanism to the step emulsification device [15]. As the fluid exits the HAR channel into the chamber, the strong constraints of the interface are released, and droplets are generated by the surface tension. In this case, the size of the droplet is determined by the channel width. That is, uniform droplets can be generated without being affected by the flow velocity when the aspect ratio (AR, the ratio of width to height) exceeds 3.5. In order to fabricate such HAR microfluidic channels, however, it requires the use of expensive deep reactive ion etching (RIE) equipment or a UV-based LIGA process [16].

In this study, we present a microfluidic device with a high-aspect-ratio (HAR) channel, which has a parallelogram cross-section, for generating monodisperse droplets. The developed HAR channel was fabricated using simple and cheap MEMS processes, such as photolithography, anisotropic KOH (potassium hydroxide) wet etching, and polydimethylsiloxane (PDMS) molding. Compared with the previously used HAR channel fabrication methods, the developed HAR channel was successfully fabricated without expensive equipment, and more importantly, the parallelogram cross-section channel structure, regarded as a very difficult structure to implement previously, was successfully integrated into a microfluidic device, particularly droplet generation configuration. In addition, compared with the above-mentioned HAR channel with a rectangular cross-section [15,16], our presented HAR channel with a parallelogram cross-section can generate more stable and smaller droplets for a wider range of flow rates. Here, we investigate the effects of the cross-sectional shape (parallelogram vs. rectangle) and height-to-width ratio (AR) of a microfluidic channel on the size and uniformity of generated droplets. To the best of our knowledge, the effect of the cross-sectional shape on the droplet generation has not been examined yet, and the developed microfluidic device is the first platform that employs the parallelogram cross-section channel in droplet production.

## 2. Materials and Methods

### 2.1. Design of a High-Aspect-Ratio (HAR) Microfluidic Channel with a Parallelogram Cross-Section

**Figure 1:**
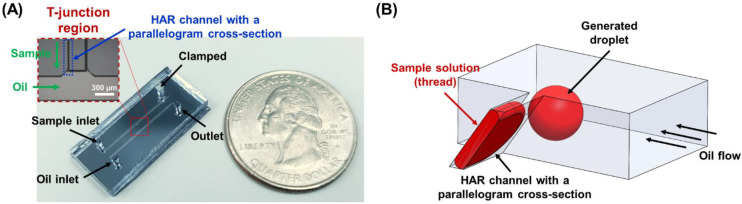
(**A**) Photographs of the developed microfluidic device comprising a HAR channel with a parallelogram cross-section. An inset shows the microscopic image of a T-junction droplet generation region in the microfluidic device where sample solution flows through the HAR channel with a parallelogram cross-section. (**B**) A schematic illustration of the HAR channel with a parallelogram cross-section in the T-junction region, which induces self-breakup droplet generation.

The developed microfluidic device is composed of two inlets for sample solution and carrier oil solution, an interconnecting channel for generating droplets at the interface of the oil inlet (T-junction droplet generation region), and an outlet for collecting the generated droplets (Figure 1). The middle interconnecting channel is designed to have a HAR geometry of a parallelogram cross-section, where its base width (W), height (H), and hypotenuse (HT) are 13.5, 51.0, and 62.4 μm, respectively, displaying aspect ratios (ARs) of 3.8 (H/W) and 4.6 (HT/W). The angle (θ) between the base width and the side length always forms 54.7° since this is the angle between (111) and (100) of single crystal Si. When the HAR channel (AR > 3.5) is utilized to provide the sample solution in the T-junction configuration, the droplet breakup mechanism is mainly dominated by the interfacial tension, and the effect of the shear stress on the droplet formation can be neglected [12,13]. The developed microfluidic device employing the HAR channel with a parallelogram cross-section enables the sample solution to self-break up into monodisperse droplets without being affected by fluid properties and flow rates.

### 2.2. Fabrication Process

**Figure 2:**
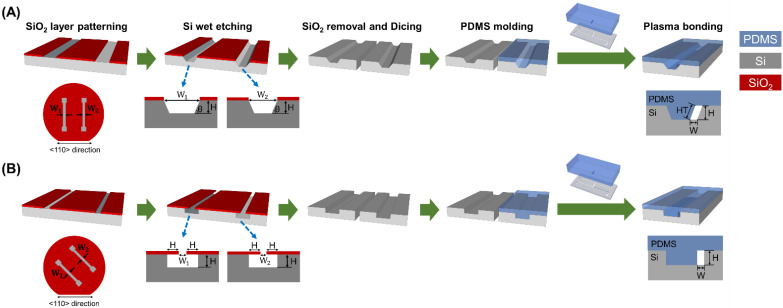
The fabrication process of the microfluidic devices consisting of a HAR channel (**A**) with a parallelogram cross-section and (**B**) with a rectangular cross-section. The Si channel and the Si master for the PDMS mold are fabricated on a single Si wafer, allowing for both the Si channel and the PDMS mold to have the same height. The channel width (W) in both parallelogram and rectangular cross-section designs was determined by an initial photomask design, resulting in W = W_1_ − W_2_. The channel height (H) can be easily controlled by adjusting the etching time.

Previously, we reported the fabrication process of a channel with a parallelogram cross-section [17,18]. Photolithography, anisotropic KOH wet etching, plasma bonding, and self-alignment between PDMS and Si were sequentially performed to fabricate channels having parallelogram and rectangular cross-sections, as shown in Figure 2. First, a SiO_2_ thin film layer of 1000 Å thickness was deposited on a (100) single crystal Si wafer using low-pressure chemical vapor deposition (LPCVD) and patterned by photolithography and RIE (reactive ion etch) (photomask designs are illustrated in Figure S1). Then, the Si wafer was anisotropically etched with KOH solution at 70 °C (etch rate: 0.66 μm/min). The Si channels and the masters for PDMS molds for parallelogram and rectangular cross-sections had the same etching depth, so it was possible to fabricate the Si channel and master for the PDMS mold in one silicon wafer. The width of the channels (W = W_1_ − W_2_), which was determined by the photomask design for Si channel width (W_1_) and master width (W_2_) for PDMS molds, was fixed to 13.5 μm, and their heights (H) were controlled by KOH anisotropic wet etching time to fabricate the channels with various ARs. A PDMS mold was replicated from the Si master, and then was self-aligned and bonded with the Si channel by oxygen plasma treatment. A small amount of methanol (or DI water) was sprayed between the Si channel and the PDMS mold to facilitate self-alignment, where the PDMS mold could be mechanically aligned to one side wall of the Si channel by hand. Finally, the methanol was evaporated on a hot plate to complete the formation of the channel, which was composed of PDMS and silicon. Since the Si channel and the Si master for the PDMS mold were fabricated on the same wafer, both had the same crystal plane and etching depth (Figure 2). In other words, the geometrical similarity (including depth) between them enabled the easy alignment. The assembled microfluidic device is illustrated in Figure 1A.

### 2.3. Droplet Generation and Analysis

Channels in all fabricated devices were coated with Aquapel^®^ (PGW LLC, Cranberry Township, PA, USA) to make their surfaces hydrophobic, followed by rinsing with nitrogen gas and carrier oil (Novec^TM^ 7500, 3M^TM^, St. Paul, MN, USA). To flow the sample solution (deionized water) into the HAR channel, one of two inlets was clamped. Oil solution was mixed with a surfactant (008-FluoroSurfactant, RAN Biotechnologies, Beverly, MA, USA) at a 1% (*w*/*w*) ratio for stable droplet generation and storage. Both sample and oil solutions were injected using syringe pumps (Fusion 200, Chemyx Inc., Stafford, TX, USA).

**Figure 3:**
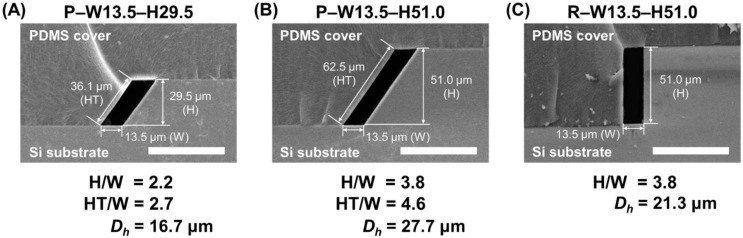
The SEM images showing the cross-section of the fabricated HAR channels. (**A**) Cross-section of a parallelogram channel with AR lower than 3.5 (AR = 2.2, P–W13.5–H29.5). (**B**) Cross-section of a parallelogram channel with AR larger than 3.5 (AR = 3.8, P–W13.5–H51.0). (**C**) Cross-section of a rectangular channel with AR larger than 3.5 (AR = 3.8, R–W13.5–H51.0) (scale bar: 100 µm).

To compare the effect of cross-sectional shapes (parallelogram or rectangle) and ARs on the droplet generation, microfluidic devices with 3 different interconnecting channel designs (P–W13.5–H29.5, P–W13.5–H51.0, and R–W13.5–H51.0; having the same channel width (W) but different shapes (P: parallelogram, R: rectangle) and heights (H), Figure 3) were characterized. The flow rate of the carrier oil solution was fixed at 400 µL/h, and droplet generation under various flow rates of the sample solution (10, 30, 50, 100, and 200 µL/h) was analyzed. For further precise comparison between P–W13.5–H51.0 and R–W13.5–H51.0, even lower flow rates of the sample solution (1, 2, and 5 µL/h) were tested. The hydraulic diameters (*D_h_*) of 3 different interconnecting channel designs were calculated from the definition of Reynolds number (*Re*) as below:

$$Re=\frac{\rho VD_{h}}{\mu }=\frac{\rho \frac{Q}{A}\frac{4A}{P}}{\mu }=\frac{4\rho }{\mu }\frac{Q}{P}\;\;\;\left(\;V=\frac{Q}{A}\;,\;\;\;D_{h}=\frac{4A}{P}\;\right)$$

where *ρ* is the fluid density, *V* is the characteristic velocity, *D_h_* is the hydraulic diameter, μ is the mean viscosity, *A* is the cross-sectional area of the flow, *P* is the wetted perimeter of the cross-section, and *Q* is the flow rate.

The developed HAR channel has a parallelogram cross-section structure, which is not symmetric, and depending on the flow direction of oil solution, the sample solution would experience different flow profiles at the interface (i.e., T-junction region). For example, when the oil solution flows towards the outlet, this carrier solution interacts with the sample solution with an angle of 54.7°, while the oil solution meets the sample solution with an angle of 125.3° when flowing towards the inlet. In order to examine the effect of the different flow profiles at the interface, the droplet production under different oil flow directions was compared (oil flow rate: 400 µL/h). In addition, flow rates of 200, 400, 600, and 800 µL/h in the oil solution were tested to investigate their influence on the produced droplet size. The flow rate of the sample solution was maintained at 50 µL/h. For this characterization, a HAR channel with a parallelogram cross-section (P–W11.8–H51.0; W: 11.8 µm, H: 51.0 µm, HT: 62.4 µm, H/W: 4.3, HT/W: 5.3) was utilized.

Droplet generation was monitored using an upright microscope (BXFM-F, Olympus, Tokyo, Japan) equipped with a high-speed camera (MIRO EX4-4096MC, Phantom, Wayne, NJ, USA). The generated droplets were collected through the outlet, and their sizes were measured by bright-field microscopy and NIH ImageJ software. Each datum shown in the manuscript was measured from at least 30 droplets.

## 3. Results and Discussion

### 3.1. High-Aspect-Ratio Microfluidic Channel with a Parallelogram Cross-Section

Figure 3 shows the scanning electron microscope (SEM) images of the fabricated channels having parallelogram and rectangular cross-sections. These cross-sectional images clearly demonstrate that the Si channel and the PDMS mold (replicated from the Si master) were perfectly aligned and bonded because of their geometrical similarity. The width of both fabricated channels was found to be 13.5 μm, which was successfully determined by the width of the initial photomask design. The height of the fabricated channels was easily controlled by adjusting the anisotropic KOH etching time. For example, channels in Figure 3A,B, both have a parallelogram cross-section and the same channel width of 13.5 μm, but exhibit different channel heights, 29.5 and 51.0 μm (H/W = 2.2 and 3.8), where a deeper channel height was achieved through the longer wet etching process. Figure 3C illustrates the cross-section of the fabricated rectangular interconnecting channel, which has the same width and height, resulting in the same AR (H/W = 3.8) compared with Figure 3B. Both channels have the same cross-sectional areas and Reynolds number for a given flow rate, but the hydraulic diameter is different. It means that the fluid would experience the different force when it flows out from the channel.

The HAR channel with a parallelogram cross-section was successfully fabricated using simple and cheap MEMS processes without requiring any expensive equipment. In addition, the parallelogram channel shape, which was challenging to implement in conventional fabrication methods, was built and integrated into the microfluidic device. To the best of our knowledge, the developed microfluidic device is the first platform that utilizes the parallelogram cross-section channel for droplet generation.

### 3.2. Characterization of Droplet Generation with Different Channel Geometries

**Figure 4:**
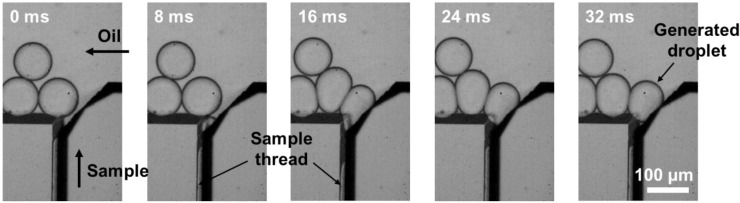
Time-lapse images displaying the droplet generation process at the T-junction region.

Time-lapse images showing the droplet generation process at the water–oil interface are illustrated in Figure 4. When the sample solution (DI water) was introduced in the HAR channel with a parallelogram cross-section, it was pressurized and formed a thin thread. As the sample solution reached close to the T-junction region, the thin thread pinched off into a droplet. Once the formed droplet was released to an oil-flowing channel, the elongated thread retracted, resumed the initial state, and repeated these steps, resulting in monodisperse droplets being produced periodically (Video S1).

**Figure 5:**
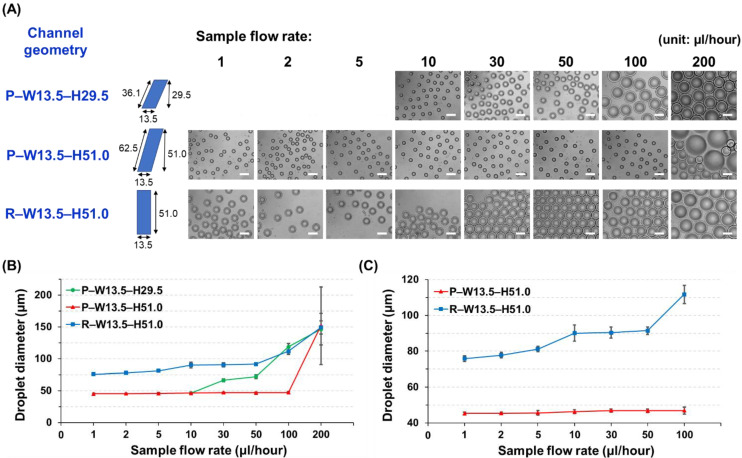
The effect of channel geometries (cross-section shape and AR) on droplet generation. (**A**) Micrographs showing the droplets produced using different channel geometries under various flow rate conditions of the sample solution. The flow rate of the oil solution was fixed at 400 µL/h (scale bar: 100 µm). (**B**,**C**) Analysis of the average droplet sizes by different geometries and sample flow rate conditions.

The effect of the channel geometries, such as cross-sectional shapes (parallelogram or rectangle) and ARs (larger or smaller than 3.5), on droplet formation was investigated. The diameters of the droplets created from all the different geometries and flow rate conditions (oil: 400 µL/h, sample: 1, 2, 5, 10, 30, 50, 100, and 200 µL/h) were measured and compared, where all measurement results are summarized in Table 1. It can be seen in Figure 5A,B that the modality of the droplet formation is different in accordance with the channel geometries. For example, when the channel structure with an AR value of less than 3.5 (P–W13.5–H29.5, AR = 2.2) was used, the droplet breakup profile followed a conventional T-junction droplet generation principle, which is governed by the viscous shearing forces between oil and sample solutions. In this case, the size of the produced droplet was strongly dependent on flow variations, where larger-diameter droplets were formed as the faster flow of the sample solution was applied (Video S2). In the P–W13.5–H29.5 channel design, the average diameter of the droplets was 46.2 µm under the sample flow rate of 10 µL/h, and then their sizes began to increase as the sample flow rates became larger, where droplets with an average diameter of 146.3 µm were created under a sample flow rate of 200 µL/h.

On the other hand, when the HAR channel structure (AR > 3.5) was employed, the interfacial tension became the dominant force for droplet generation, enabling monodisperse droplets to be formed regardless of flow fluctuations. In the P–W13.5–H51.0 channel design (AR = 3.8), the average sizes of the droplets were kept almost consistent, where less than 1.5% size variation was observed between sample flow rates ranging from 10 to 100 µL/h (Table 1, Figure 5B, and Video S3). In the R–W13.5–H51.0 channel design (AR = 3.8), the average diameters of the droplets created under the sample flow rate conditions ranging from 10 to 50 µL/h were also almost uniform (Table 1, Figure 5B, and Video S4), although this rectangular cross-section design had a smaller flow rate interval for inducing the monodisperse droplet generation than the parallelogram cross-section design (P–W13.5–H51.0).

To further analyze the effect of cross-section shapes, even lower flow rates (1, 2, and 5 µL/h) of sample solutions were applied, and the droplet production profiles were compared between the P–W13.5–H51.0 and R–W13.5–H51.0 channel designs. As shown in Figure 5C, the parallelogram cross-section design (P–W13.5–H51.0) was able to produce almost uniform-sized droplets irrespective of the flow rate changes even in this lower range. However, the average droplet diameters created from the rectangular cross-section design (R–W13.5–H51.0) showed the dependency on the flow variations (e.g., 75.8 µm at 1 µL/h and 81.2 µm at 5 µL/h), indicating that the viscous shearing force was the dominant force in the lower range, and uniform-sized droplets could not be implemented. In addition, the hydraulic diameters (*D_h_*) of three different channel designs were calculated and compared (Table 1 and Figure 3), where the channel design with a larger hydraulic diameter (P–W13.5–H51.0, *D_h_* = 27.7 µm) showed the generation of monodisperse droplets within a wider range of flow rates against other designs (P–W13.5–H29.5, *D_h_* = 16.0 µm, R–W13.5–H51.0, *D_h_* = 21.3 µm). From the above characterization results of droplet generation among three different geometries, our developed HAR channel with a parallelogram cross-section clearly demonstrated its outperforming capability, which could generate uniform-sized droplets independent of flow variation within a wider range (flow rate range for uniform-sized droplet generation: the developed HAR parallelogram channel = 1 ~ 100 µL/h vs. the HAR rectangular channel = 10 ~ 50 µL/h).

Another interesting feature of the parallelogram cross-section design is that the produced droplets have smaller sizes compared with the rectangular cross-section design. In the P–W13.5–H51.0 and R–W13.5–H51.0 channels, both designs have the same channel width and cross-sectional area, so they would have to generate similar sizes of droplets under the same flow conditions. However, when comparing the droplet sizes created under the same flow conditions, the parallelogram cross-section design (P–W13.5–H51.0) exhibits smaller sizes all the time. This size difference would be mainly due to different cross-sectional shapes, particularly the hypotenuse of the parallelogram. When the sample solution is filled inside the P–W13.5–H51.0 channel, the channel surface that interacts with the solution thread to produce droplets is the hypotenuse of the parallelogram, not its height. Thus, although the parallelogram cross-section channel has the same channel height (same AR) and cross-sectional area compared with the rectangular cross-section channel, this design can provide a larger surface interaction (the length of the hypotenuse = the channel height/sin (54.7°) = 1.23 × the channel height = 62.5 µm), which would result in a larger interfacial tension. The larger interfacial tension might induce more frequent droplet breakups along the parallelogram cross-section channel, and this would be the main reason for smaller droplets being produced with higher generation frequencies (Videos S3 and S4). In addition, the larger interfacial tension deriving from the interaction with a longer hypotenuse would be the main contributor for a wider range of flow variation that can create the uniform-sized droplets in the parallelogram cross-section channel. Numerical simulations based on the volume of fluid were also conducted to clearly understand the effect of the channel cross-section geometries on the droplet generation, where the difference in the droplet breakup mechanism as well as the sample flowing position among three different designs was observed (Supplementary Information, Figures S2 and S3).

### 3.3. Effect of the Carrier Oil on Droplet Generation

**Figure 6:**
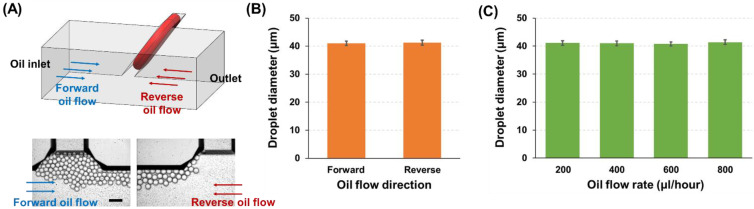
The effect of the carrier oil flow on droplet generation. (**A**) Illustration and microscopic images showing the droplet generation under two different flow directions of the oil solution (forward: towards a device outlet; reverse: towards an oil inlet) (scale bar: 100 µm). (**B**) Comparison of the average droplet sizes produced under two different oil flow conditions (sample flow rate: 50 µL/h; oil flow rate: 400 µL/h). (**C**) Analysis of the average droplet sizes formed under various oil flow rates (200, 400, 600, and 800 µL/h, sample flow rate was fixed at 50 µL/h).

As the developed HAR channel has the parallelogram structure, the channel cross-section is tilted at an angle of 54.7° to the bottom surface. This asymmetric channel structure can cause different flow profiles at a sample (water)–oil interface when the oil flow directions change in the T-junction. To confirm whether the droplet generation was affected by the oil flow directions, the flow rates of the sample and oil solutions were fixed at 50 and 400 µL/h, respectively, and the droplet formation was monitored only by varying the oil flow direction (Figure 6A). As can be seen in Figure 6B and Video S5, no significant difference in droplet generation (droplet diameter: 41.0 ± 0.8 µm vs. 41.2 ± 0.9 µm, corresponding to oil flow direction towards outlet (forward flow) vs. oil inlet (reverse flow)) was observed from different oil flow directions (i.e., different flow profiles). In addition, the effect of oil flow speed on the droplet production was tested (Figure 6C and Video S6), where no significant difference was found from various oil flow rates ranging from 200 to 800 µL/h (droplet diameter: 41.1 ± 0.8, 41.0 ± 0.8, 40.8 ± 0.7, and 41.4 ± 0.8 µm at oil flow rates of 200, 400, 600, and 800 µL/h, respectively). These results from different oil flow conditions verify that the droplet generation mechanism in the developed HAR parallelogram channel is mainly dominated by the interfacial tension, which induces self-breakup of droplets and is not affected by flow variation.

One thing to note here is that the droplet size (diameter: 41.0 µm) generated from the HAR parallelogram channel having a 11.8 µm width is smaller than the previously used channel design (P–W13.5–H51.0) comprising a 13.5 µm width (droplet diameter: 46.8 µm). Since both channel designs had the same height and hypotenuse, the different droplet sizes were mainly derived from different lengths of the channel widths. This result clearly demonstrates the capability of the developed HAR parallelogram channels to control the droplet sizes by simply adjusting the channel width.

## 4. Conclusions

In this paper, we developed the microfluidic device comprising the high-aspect-ratio (HAR) channel with a parallelogram cross-section for generating monodisperse droplets. The developed device was fabricated using simple and cheap MEMS processes, where the HAR parallelogram channel geometry, very difficult to implement previously, was successfully created and integrated into the microfluidic device without requiring high-cost equipment and processes. The droplet generation was characterized using the developed channel design, and the effect of the channel geometries including the aspect-ratio and cross-sectional shape was investigated. In addition, the performance of the developed parallelogram channel was compared with that of a previously developed rectangular channel. The results successfully demonstrated the outperforming capability of the developed HAR parallelogram channel, where uniform-sized droplet generation was confirmed in a wider range of flow variation. To the best of our knowledge, the developed microfluidic device is the first platform that utilizes a parallelogram cross-section channel for generating droplets.

## Figures and Tables

**Table 1:** Average diameter of droplets generated from different channel geometries and flow rate conditions.

		Sample Flow Rate (µL/h)							
		1	2	5	10	30	50	100	200
**Channel geometry**	**P–W13.5–H29.5** **(parallelogram,** **AR < 3.5, *D_h_* = 16.7 μm)**				46.2 ± 1.4	66.3 ± 2.3	72.0 ± 2.9	118.5 ± 5.6	146.3 ± 24.7
	**P–W13.5–H51.0** **(parallelogram,** **AR > 3.5, *D_h_* = 27.7 μm)**	45.3 ± 0.7	45.3 ± 0.7	45.6 ± 1.3	46.3 ± 1.0	46.9 ± 0.9	46.8 ± 0.9	46.9 ± 2.0	151.9 ± 60.9
	**R–W13.5–H51.0** **(rectangle,** **AR > 3.5, *D_h_* = 21.3 μm)**	75.8 ± 1.6	77.8 ± 1.6	81.2 ± 1.5	90.0 ± 4.6	90.3 ± 3.2	91.4 ± 2.1	111.7 ± 5.2	149.2 ± 10.5

(unit: µm).

## Supplementary Materials

The following supporting information can be downloaded at: https://www.mdpi.com/article/10.3390/bios12020118/s1, Figure S1. Photomask designs of microfluidic devices; Supplementary Information. Numerical simulations of the droplet generation in channels with parallelogram and rectangular cross-section; Figure S2. Contours of the void fraction of the dispersed phase in the cross plane; Figure S3. Contours of the void fraction of the dispersed phase in the top plane of the interconnecting channel; Video S1. Droplet generation at the water–oil interface (T-junction region) displaying the self-breakup into a monodisperse droplet; Video S2. Droplet generation in the P–W13.5–H29.5 channel (parallelogram, AR < 3.5) under various flow rate conditions; Video S3. Droplet generation in the P–W13.5–H51.0 channel (parallelogram, AR > 3.5) under various flow rate conditions; Video S4. Droplet generation in the R–W13.5–H51.0 channel (rectangle, AR > 3.5) under various flow rate conditions; Video S5. The effect of oil flow direction on the droplet generation in the HAR channel with a parallelogram cross-section (AR > 3.5); Video S6. The effect of oil flow rates on the droplet generation in the HAR channel with a parallelogram cross-section (AR > 3.5).
