# Supplementary material for: High-Aspect-Ratio Microfluidic Channel with Parallelogram Cross-Section for Monodisperse Droplet Generation
Source: Biosensors (Basel). 2022 Feb 14;12(2):118. doi: 10.3390/bios12020118 (PMC8869682; doi:10.3390/bios12020118)
Supplement: Supplementary file 1 [file biosensors-12-00118-s001.zip › biosensors-1565367-supplementary.pdf]

biosensors

High-Aspect-Ratio Microfluidic Channel with Parallelogram Cross-Section for Monodisperse Droplet Generation

Hyeonyeong Ji [1], Jaehun Lee [2], Jaewon Park [3], Jungwoo Kim [1], Hyun Soo Kim [4,*] and Younghak Cho [1,*]

1 Department of Mechanical System Design Engineering, Seoul National University of Science & Technology, Seoul 01811, Korea; jhy8718@naver.com (H.J.); kimjw@seoultech.ac.kr (J.K.)
2 Korea Institute of Machinery and Materials, Daegu Research Center for Medical Devices and Rehabilitation, Daegu 42994, Korea; ljh30226@naver.com
3 School of Microelectronics, Southern University of Science and Technology, Shenzhen 518055, China; jwpark@sustech.edu.cn
4 Department of Electronic Engineering, Kwangwoon University, Seoul 01897, Korea
* Correspondence: hyunsookim@kw.ac.kr (H.S.K.); yhcho@seoultech.ac.kr (Y.C.)

**Figure S1**	Photomask designs for microfluidic devices
**Supplementary Information**	Numerical simulations of the droplet generation in channels with parallelogram and rectangular cross-section
**Figure S2**	Contours of the void fraction of the dispersed phase in the cross plane
**Figure S3**	Contours of the void fraction of the dispersed phase in the top plane of the interconnecting channel

Citation: Ji, H.Y.; Lee, J.; Park, J.; Kim, J.; Kim, H.S.; Cho, Y. High-Aspect-Ratio Microfluidic Channel with Parallelogram Cross-Section for Monodisperse Droplet Generation. *Biosensors* **2022**, *12*, 118. https://doi.org/10.3390/bios12020118

Received: 5 January 2022
Accepted: 8 February 2022
Published: 14 February 2022

Publisher's Note: MDPI stays neutral with regard to jurisdictional claims in published maps and institutional affiliations.

Copyright: © 2022 by the authors. Licensee MDPI, Basel, Switzerland. This article is an open access article distributed under the terms and conditions of the Creative Commons Attribution (CC BY) license (https://creativecommons.org/licenses/by/4.0/).

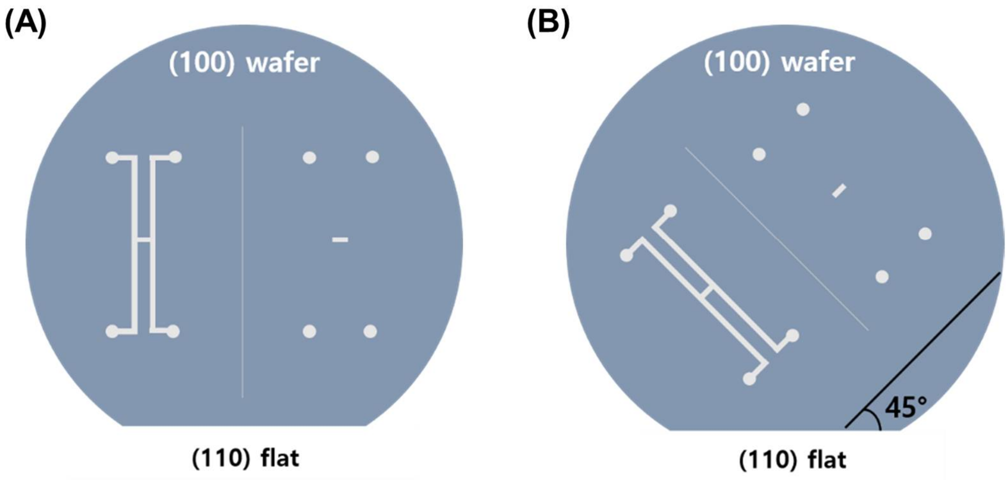

Figure S1. Photomask designs for a microfluidic device with (**A**) a parallelogram cross-section and (**B**) a rectangular cross-section.

Supplementary Information

In this paper, numerical simulations were performed in order to obtain further understanding of the droplet generation in channels with a parallelogram and rectangular cross-section by using a commercial software package, Ansys Fluent. The volume of the fluid model was employed to track the volume fraction of the dispersed (i.e., droplet) and continuous phases. The methods considered here are similar to those in Xu et al.

(2018) [1]. In the simulations, the channels (R–W25–H50, R–W25–H100, and P–W25–H100) have the same aspect ratios considered in the present experiments, although the height of their main channels (100 μm) is smaller than that in experiments due to computational efficiency. The numbers of grid points are about 1.5 million. As the boundary conditions, the mass flow rates in the main and branch channels were 400 μL/hour and 30 μL/hour, respectively. Additionally, the pressure condition was adopted in the outlet of the channel.

Figure S2 shows the void fraction of the dispersed phase in the cases considered in this study. Cases (A) and (B) are the rectangular shape of the interconnecting channel with aspect ratios of 2 and 4, respectively. Case (C) is the parallelogram shape of the interconnecting channel with an aspect ratio of 4. Figure S2 shows that the droplet breakup depends on the shape of the interconnecting channel. The size of the droplets in case (C) is smaller than that in case (B). This result agrees with that in Figure 5. In the rectangular interconnecting channel, the droplet breakup occurs near the junction, but in the parallelogram interconnecting channel, it does inside the interconnecting channel. Therefore, the droplet in the parallelogram interconnecting channel is less affected by the main channel. The difference in the droplet breakup could provide some explanation in Figure 5.

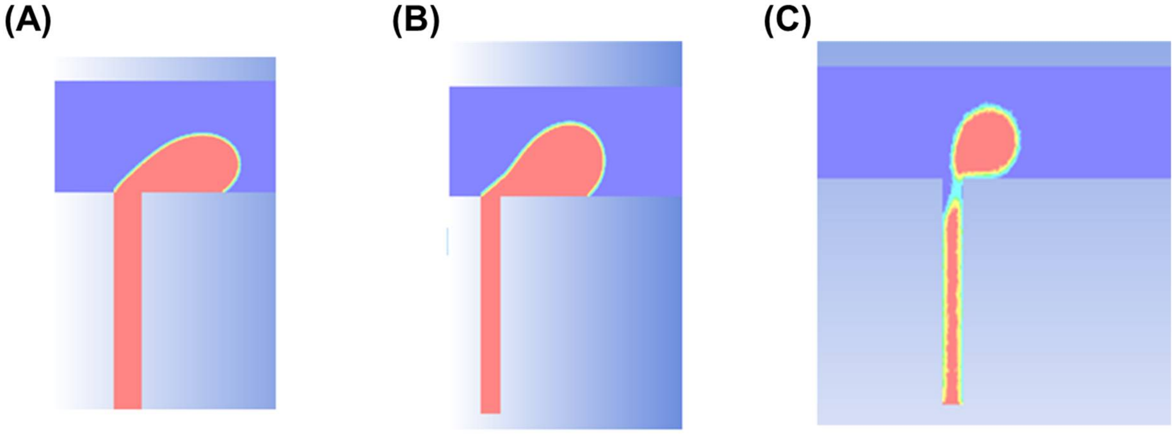

Figure S2. Contours of the void fraction of the dispersed phase in the cross plane: (**A**) R–W25–H50, (**B**) R–W25–H100, and (**C**) P–W25–H100. In (A) and (B), y = 0.5 HM, whereas in (**C**), y = 0.75 HM. Here, y is the vertical position where the cross plane is formed, and HM is the height of the main channel. Red and blue indicate the dispersed and continuous phases, respectively.

Figure S3 shows the void fraction of the dispersed phase in the cases considered in this study. In Figure S3, the dispersed phase flowing in the interconnecting channel depends on the aspect ratio. At a small aspect ratio, the dispersed-phase larger aspect ratio, the dispersed phase fills the interconnecting channel (see Figure S2A), but at a large aspect ratio, the dispersed phase flowing in the interconnecting channel is concentrated in the center or corner (see Figures S2B and S2C). Therefore, at a large aspect ratio, the necking of the dispersed phase is found in the interconnecting channel, which was also observed in Xu et al. (2018) (for reference, see Figure 3 in Xu et al. (2018) [1]). It means that even in the parallelogram shape of the interconnecting channel, the droplet breakup is closely related to the surface tension force.

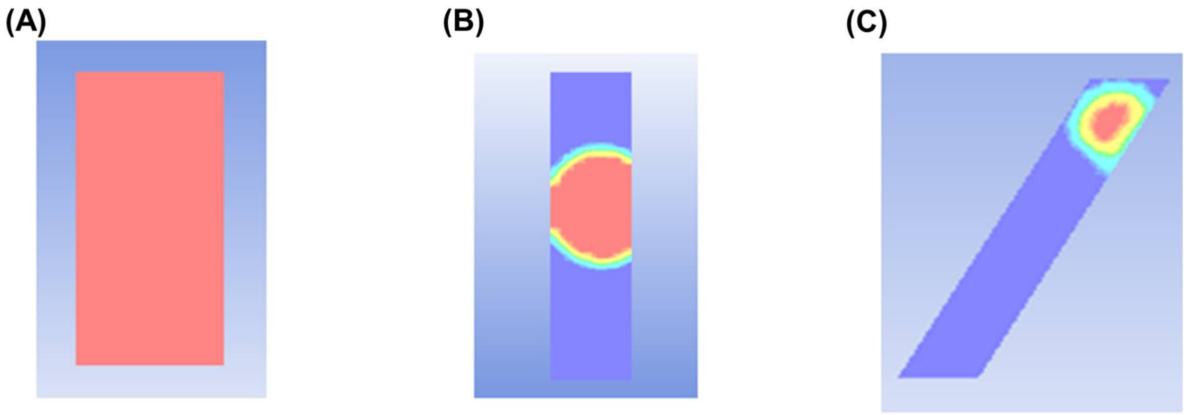

Figure S3. Contours of the void fraction of the dispersed phase in the top plane of the interconnecting channel: (**A**) R–W25–H50, (**B**) R–W25–H100, and (**C**) P–W25–H100. Red and blue indicate the dispersed and continuous phases, respectively.

Reference

1. Xu, X.; Yuan, H.; Song, R.; Yu, M.; Chung, H.Y.; Hou, Y.; Shang, Y.; Zhou, H.; Yao, S. High aspect ratio induced spontaneous generation of monodisperse picolitre droplets for digital PCR. *Biomicrofluidics* **2018**, *12*, 014103.
